# A closer look at the eyelashes!

**DOI:** 10.11604/pamj.2019.33.308.18232

**Published:** 2019-08-20

**Authors:** Kawtar Belkhadir, Ouafae Cherkaoui

**Affiliations:** 1 Mohammed V University, Rabat, Morocco; 2 Hôpital des Spécialités, Ophthalmology Department A, Rabat, Morocco

**Keywords:** Blepharitis, phtirius pubis, infectiology

## Image in medicine

A 7-year-old child presented with bilateral anterior blepharitis. He had no medical history. The careful examination of the eyelashes found several lice attached to them with their ovoid nits. The parasitological examination revealed a phtirius pubis. Screening of other family members did not find similar effects. The treatment consisted in the removal of all the lesions and their nits. Because mercury-based ointments were unavailable in our country, we have advocated rigorous hygiene of the eyelids, and the use of antibiotic ointment to smother the lice and their nits. The mode of transmission in this case is unknown. There was no evidence of ill-treatment or poor hygiene in the family. The evolution after treatment has been favorable.

**Figure 1:**
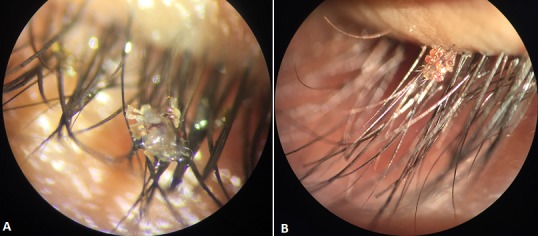
A) phtirius pubis glued to the eyelashes (magnification 40); B) phtirius pubis glued to the eyelashes (magnification 25)

